# Unilateral proptosis and blindness caused by meningioma in a patient treated with cyproterone acetate

**DOI:** 10.3205/oc000027

**Published:** 2015-06-17

**Authors:** Celine Sys, Philippe Kestelyn

**Affiliations:** 1 Ghent University Hospital, Department of Ophthalmology, Ghent, Belgium

## Abstract

Cyproterone has antiandrogenic, antigonadotropic, and progestagenic activity. High-dose preparations are used for treatment of prostate cancer and for treatment of hypersexuality. We describe a patient who was referred to our clinic with slowly progressive unilateral proptosis and blindness of the left eye. He had been treated with high-dose cyproterone actate (CPA) for 23 years. An obvious proptosis and exodeviation of his left eye was noted on ophthalmic examination. Fundoscopy showed left optic atrophy. The literature suggests a link between long-term high-dose exogenous progesterone agonist exposure and the progression and/or development of meningioma. MRI of the brain was performed and revealed multiple meningiomas. One large meningioma located in the anterior temporal lobe extended into the left orbit and caused the proptosis and blindness. Treatment with CPA was stopped and follow-up imaging 11 months later showed a significant decrease in size of the largest meningiomas.

## Introduction

Cyproterone is a derivative of progesterone with antiandrogenic, antigonadotropic, and progestagenic activity. High dose preparations are indicated for treatment of prostate cancer (200–300 mg per day) and for treatment of hypersexuality (50–600 mg per day). Cyproterone blocks the binding of dihydrotestosterone to prostatic cancer cells and exerts negative feedback on hypothalamic-pituitary axis by inhibiting luteinizing hormone secretion leading to decreased testosterone production.

Lower dose (2 mg) CPA is available for use in women in combination with ethinylestradiol (0.035 mg) for the treatment of severe acne and hirsutism.

## Case description

A 42-year-old male was referred to the department of ophthalmology with slowly progressive proptosis of the left eye. The patient had an intellectual disability caused by perinatal asphyxia and he was treated with cyproterone acetate (CPA) 100 mg/day for 23 years to reduce his undesirable sexual behavior. 

**Figure 1:**
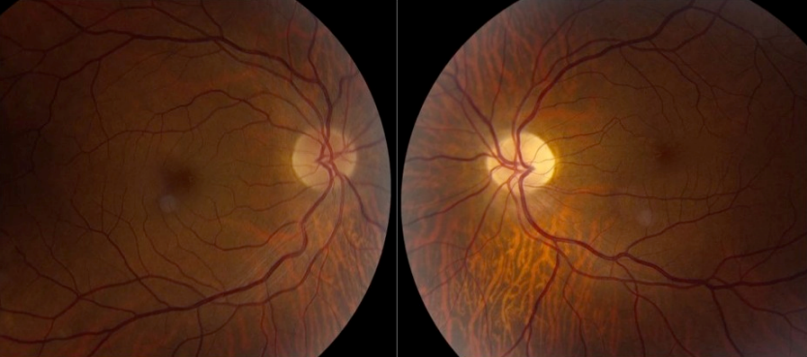
Pale optic disc in the left eye

On examination, his best-corrected visual acuity was 6/9.5 in the right eye and there was no perception of light in the left eye. An obvious proptosis and exodeviation of his left eye was noted. Fundoscopy was unremarkable in the right eye and showed optic atrophy in the left eye (Figure 1 (Fig. 1)).

**Figure 2:**
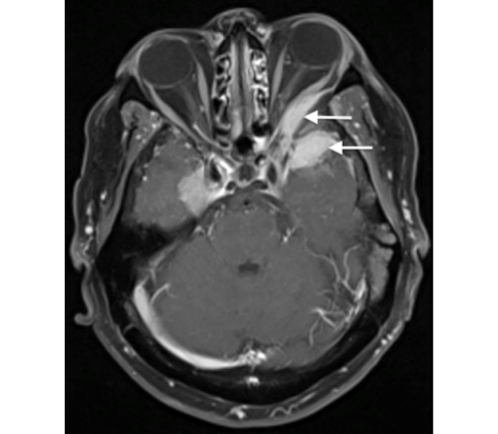
Meningioma located in the anterior temporal lobe and extending into the left orbit causing proptosis of the left eye and compression of the left optic nerve.

There is a positive correlation between the long-term use of high-dose CPA and the development of new meningiomas and/or progression of existing meningiomas. MRI of the brain and the orbit was performed and revealed 11 meningiomas. One large meningioma located in the anterior temporal lobe extended into the left orbit and caused proptosis and compression of the optic nerve leading to blindness of the left eye (Figure 2 (Fig. 2)).

**Figure 3:**
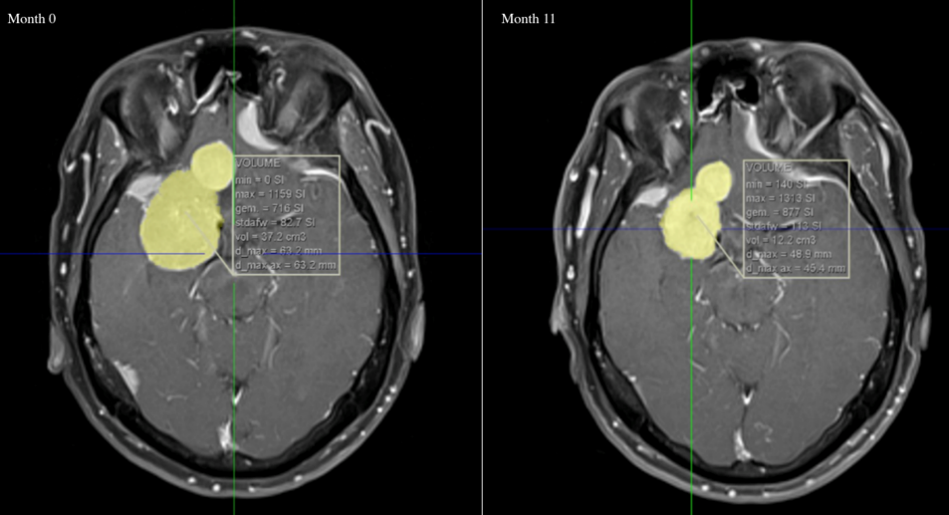
Large lobulated meningioma in the right anterior fossa. The total volume was 37.2 cm^3^ at presentation. Eleven months after discontinuation of CPA total volume was reduced to 12.2 cm^3^.

CPA was discontinued as regression of meningiomas after discontinuation of CPA has been described. On follow-up imaging 11 months after cessation of CPA, a regression with 25 cc of the volume of the largest meningioma was recorded (Figure 3 (Fig. 3)).

## Discussion

Froelich et al. reported in 2008 that long-term treatment with high daily doses of CPA could be responsible for the development of meningiomas. He described multiple meningiomas in 9 female patients treated with CPA (50 mg per day). Six patients were followed radiologically for a period exceeding 5 months (8 to 81) before treatment withdrawal. A significant increase in tumor size and/or the development of new lesions was observed in all cases [1].

A large controlled population-based study using data from the Health Improvement Network UK primary care database confirmed a significantly increased risk of meningioma in male patients taking high-dose CPA. However, these risk estimates were based on only four cases of meningioma. There was no significant correlation between meningioma and low-dose CPA use in female patients [2].

Nevertheless, the hypothesis that exposure to high-dose CPA increases the risk of meningioma is supported by another large retrospective cohort study performed in a Spanish primary care database (BIFAP). Among 2,474 users of high-dose CPA four meningioma cases were identified, resulting in an incidence rate which was significantly higher than that observed among the non-users and among users of low-dose CPA. After adjusting for age and gender, patients exposed to high-dose CPA showed an increased risk of meningioma of 11.4 (95% CI 4.3–30.8) as compared to non-users [3].

One case report of Gonçalves et al. reports a rapid regression of an incidental meningioma after discontinuation of a 10-year CPA treatment. Our case report confirms that finding. We noted a regression of the largest meningiomas up to 25 cc 11 months after cessation of high dose CPA treatment for 23 years. 

## Conclusion

This case report emphasises the importance of general anamnesis in every patient. Always ask about general health and drug intake. 

Consider the potential risk of a meningioma in patients receiving long-term high-dose CPA. Patients with existing meningioma or a history of meningioma should not be prescribed high-dose (>25 mg/day) CPA. If a diagnosis of meningioma is made in a patient on CPA, it is important to realise that withdrawal of CPA may induce regression of existing meningiomas.

## Notes

### Competing interests

The authors declare that they have no competing interests.
